# The New Medical Code

**Published:** 1883-06

**Authors:** 


### The New Medical Code.

There is no denying the fact that the controversy in the State of New York over the new medical code is becoming acrimonious and bitter. This is greatly to be regretted, for it makes any compromise difficult, and tends to prevent the peaceable acceptance of the result by the minority, should the question at any time be definitely decided. An appeal to the prejudices of some men is far more productive of results than an address to their reason, hence the danger that the question will not be dispassionately settled, and that extreme partizans will take advantage of this fact to remain recalcitrant. All classes of medical men will readily declare themselves in favor of true progress, but will honestly differ in their estimate of so decided a step as that proposed by the advocates of the new code. Upon its face, the question of permitting a regular practitioner to give his advice in a critical case to any patient who may desire it, does not seem very alarming. But if this carries with it an acknowledgment of all the various isms and pathies by which medicine is vexed, it is well to think twice about it. If medical science be a science at all, there can be but one school, and the practitioners of that school are not only at liberty to use, but are in duty bound to employ every remedy which proves efficacious in the control of disease. It matters not whence the drug is derived, it belongs to every medical man. That physician who refuses to employ a remedy because it was first brought into favor by some irregular practitioner, is recreant to his duty. The virtues of cold water were known to medicine long before Hydropaths endeavored to found a school upon its exclusive use. It ought not to be a crime for an educated physician, when a human life is at stake, to point out to a specialist, in the consultation room, that a good thing may be abused. But the acknowledging of a cold water fanatic as a medical man is quite another matter. Whatever is done should be by the profession as a whole, and the disrupting of fraternal professional relations is a thing to be deplored. Therefore, the proposal to refer the whole matter to the American Medical Association, provided there can be a full and fair expression of professional preference, would seem to be the easiest method of settling the question. Meantime the Homœopaths, the most numerous of all the outside medical sects, are standing by, amused spectators of the struggle.—*Independent Practitioner*.

